# Oridonin Relieves Angiotensin II-Induced Cardiac Remodeling via Inhibiting GSDMD-Mediated Inflammation

**DOI:** 10.1155/2022/3167959

**Published:** 2022-03-14

**Authors:** Shuang Lin, Shanshan Dai, Jiahui Lin, Xiaohe Liang, Weiqi Wang, Weijian Huang, Bozhi Ye, Xia Hong

**Affiliations:** ^1^The Key Laboratory of Cardiovascular Disease of Wenzhou, Department of Cardiology, The First Affiliated Hospital of Wenzhou Medical University, Wenzhou, Zhejiang, China; ^2^Department of Emergency, The First Affiliated Hospital of Wenzhou Medical University, Wenzhou, Zhejiang, China; ^3^Wenzhou Medical University, Wenzhou, Zhejiang, China; ^4^The Key Laboratory of Cardiovascular Disease of Wenzhou, Department of Cardiac Care Unit, The First Affiliated Hospital of Wenzhou Medical University, Wenzhou, Zhejiang, China

## Abstract

Myocardial remodeling is one of the main lesions in the late stage of chronic heart failure and seriously affects the prognosis of patients. Continuous activation of the renin-angiotensin-aldosterone system (RAAS) contributes to the development of myocardial remodeling greatly, and angiotensin II (Ang II), its main constituent, can directly lead to cardiac remodeling through an inflammatory response and oxidative stress. Since Ang II-induced myocardial remodeling is closely related to inflammation, we tried to explore whether the anti-inflammatory drug oridonin (Ori) can reverse this process and its possible mechanism. Our study investigated that hypertrophy and fibrosis can be induced after being treated with Ang II in cardiomyocytes (H9c2 cells and primary rat cardiomyocytes) and C57BL/6J mice. The anti-inflammatory drug oridonin could effectively attenuate the degree of cardiac remodeling both in vivo and vitro by inhibiting GSDMD, a key protein of intracellular inflammation which can further activate kinds of inflammation factors such as IL-1*β* and IL-18. We illustrated that oridonin reversed cardiac remodeling by inhibiting the process of inflammatory signaling through GSDMD. After inhibiting the expression of GSDMD in cardiomyocytes by siRNA, it was found that Ang II-induced hypertrophy was attenuated. These results suggest that oridonin is proved to be a potential protective drug against GSDMD-mediated inflammation and myocardial remodeling.

## 1. Introduction

Ang II, the main component of the RAAS, contributes to numerous cardiac pathophysiological processes. Sustained stimulation of Ang II results in excessive vasoconstriction, abnormal hypertrophy, and fibrosis in both the vasculature and cardiac tissues [1, 2, 3]. Many studies have expounded and proved that inflammation is involved and plays a vital role in Ang II-induced myocardial remodeling [4, 5, 6]. Monocytes can be activated by various harmful stimuli, resulting in the release of kinds of cytokines. Proliferation and migration of monocytes also induce inflammatory reaction, leading to interstitial fibrosis, ventricular remodeling, and eventually decreased systolic function [7]. Further investigations show that Ang II-induced myofibroblast differentiation was obstructed in NLRP3 knockout cardiac fibroblasts. Furthermore, Ang II-induced myocardial fibrosis was alleviated in NLRP3^−/−^mice, with no impact on hypertension or cardiac hypertrophy [8].

GSDMD is known as the key protein of cellular inflammatory necrosis. Following the NLRP3 inflammasome priming and the cleavage of caspase-1, GSDMD-N gathers to form pores in the cell membrane [9, 10, 11]. These pores destroy the integrity of cell membrane, causing cell swelling and rupture. The process was accompanied with the maturation and the release of intracellular interleukin-1*β* (IL-1*β*) and interleukin-18 (IL-18), which boosts the following inflammatory response [12]. Current researches suggest that GSDMD-mediated inflammation mainly occurs in innate immune cells, and some studies have shown that it exists in certain kinds of tumor cells as a protective mechanism [13, 14, 15]. A study by Zheng et al. suggests that NLRP3 inflammasome mediates the development of atherosclerosis [16]. Meanwhile, Sokolova et al. found that inhibiting NLRP3 can effectively improve ventricular remodeling and cardiac function [17]. Therefore, anti-inflammatory therapy can be regarded as an important mean to prevent and treat ventricular remodeling. Application of anti-chronic inflammatory drugs combined with conventional therapeutic agents is supposed to be a potential direction for the effective treatment of ventricular remodeling.

Oridonin is a kind of huperzine and diphenol compound extracted from the leaves of Rabdosia rubescens of the genus Rabdosia in the family Labiatae. Oridonin has been found to have strong anti-inflammatory [18, 19, 20] and growth inhibitory effects on multiple types of cancer cells [21]. Oridonin also shows antiviral effect [22]. Yang et al. demonstrated that oridonin can inhibit the NLRP3 inflammasome and NF-*κ*B pathways [23]. Therefore, we hypothesized that the proinflammatory effect of Ang II can be antagonized by oridonin, and if present, we would map the potential protective mechanism out.

## 2. Material and Methods

### 2.1. Cell Culture and Treatments

The H9c2 rat cardiomyocyte line was purchased from the Shanghai Institute of Biochemistry and Cell Biology (Shanghai, China). Cardiomyocytes were isolated from 1-to 3-day-old neonatal Sprague Dawley (SD) rats referring to previous experimental methods [24, 25, 26]. Cells were cultured in Dulbecco's modified Eagle's medium (DMEM, Gibco, USA) containing 4.5 g/L d-glucose, supplemented with 10% fetal bovine serum (FBS, Gibco, USA), 100 U/mL penicillin, and 100 U/mL streptomycin in a circumstance of 5% CO_2_ at 37°C. When density is up to 70%, cells were starved with serum-free medium for 2 h before treatment. Cells were pretreated with oridonin at test concentrations (Selleck, S2335, dissolved in DMSO) 1 h before incubation with Ang II (1 *μ*M, Aladdin, A107852) for 24 h. DMSO solution (1‰) was used as the vehicle control.

### 2.2. Animals

Thirty-two wild-type (WT) C57BL/6J mice were utilized in this research. The mice were fed a standard rodent diet at ordinary temperature in a 12 : 12 h light/dark cycle circumstance and acclimated to the laboratory for at least 1 week before initiating studies. Then, repartition the mice into four equal groups randomly: (1) subcutaneous injection of normal saline (NS, control, *n* = 8); (2) subcutaneous injection of Ang II 1.4 mg/kg/d for a period of 4 weeks and intraperitoneal injection of normal saline during the final week (Ang II+NS, *n* = 8); (3) subcutaneous infusion with Ang II 4 weeks and intraperitoneal injection of oridonin 10 mg/kg/d during the final week (Ang II+oridonin, 10 mg/kg, *n* = 8); and (4) mice infused with Ang II and a higher dosage of oridonin (Ang II+oridonin, 15 mg/kg, *n* = 8). Systolic blood pressure (SBP) of each mouse was measured by a tail-cuff pressure analysis system (BP-98A, Softron, Japan) without anesthesia. Doppler echocardiography was performed one day before killing to detect cardiac function. Four weeks later, the mice were sacrificed; their body weights were measured and recorded. The blood specimens and cardiac tissues were collected for subsequent experimental analysis at the same time.

All animal experiments were strictly performed under directives in the Guidelines for the Care and Use of Laboratory Animals (US National Institutes of Health) and Laboratory Animal Ethics subject to examination and supervision by Animal Experimental Ethical Inspection of Laboratory Animal Centre, Wenzhou Medical University.

### 2.3. siRNA Knockdown

A sequence of small interfering (siRNA) was used to knockdown *GSDMD* in H9c2 cells (the target sequence of si-*GSDMD*: GTCAAGTCTAGGCCAGAAA). The negative control (NC) siRNA and transfection reagents were constructed and prepared by RiboBio (Guangzhou, China). We chose the concentration of 50 *μ*M of siRNAs to incubate H9c2 cells for 48 h for more effective transfection. Effectiveness of gene silencing is further detected by western blotting.

### 2.4. Cell Viability Assays

A suitable concentration of cell suspension was uniformly planted in 96-well plates at 100 *μ*l per hole, and then, cells were incubated with concentration gradient of oridonin for 24 h. Cell counting kit-8 (CCK-8, Beyotime, C0037) was applied to estimate cell viability, which was performed under the direction of the manufacturer's protocol. All data came from three independent trials.

### 2.5. Western Blot Analysis

After choosing RIPA buffer (Solarbio, R0010) to lyse cells, we then centrifuged the turbid liquid and collected the supernatant. Protein samples were quantified to 20 *μ*g by a BCA protein assay kit (Solarbio, PC0020), then loaded in SDS-PAGE for further separation and later transferred onto PVDF membranes. The PVDF membranes were blocked with 5% fat-free milk at room temperature for 1 h. After being washed 3 times, the membranes were soaked in primary antibodies against GSDMD (Santa Cruz, sc-393581, 1 : 200), MyHC (Abcam, ab50967, 1 : 1000), NLRP3 (Abcam, ab263899, 1 : 1000), IL-1*β* (Abcam, ab283818, 1 : 1000), and IL-18 (Abcam, ab191860, 1 : 1000) at 4°C overnight. The membranes were subsequently reprobed with GAPDH (Cell Signaling, 2118S, 1 : 1000) for standardization. After being washed three times, they were incubated in anti-mouse IG-HRP (Beyotime, A0216) or anti-rabbit IG-HRP-(Beyotime, A0208) secondary antibodies for 1 h. Finally, visualizations of proteins by High-sig ECL Substrate (Bio-Rad, USA) were analyzed with ImageJ software.

### 2.6. Real-Time PCR Analysis

The high-quality RNA from cardiomyocytes was extracted by TRIzol Reagent according to the manufacturer's protocol (Thermo Fisher Scientific, 15596026). And cDNA was synthesized with the RevertAid First Strand cDNA Synthesis Kit (Thermo Fisher Scientific, K1622). cDNA was amplified by real-time RT–polymerase chain by SYBR (Takara Biotechnology, RR037A) performed on ABI7500 platform. All results were standardized by *GAPDH*. Primers used in real-time PCR are shown in Table 1.

### 2.7. H&E and Sirius Red Staining

Fresh myocardial tissues were taken after mice were killed. Put the specimens immediately into 4% paraformaldehyde at least 8 h to fix, then dehydrate in graded alcohol and hyalinize in xylene, and finally embed them in paraffin in accordance with standard procedures. The specimens were made into 5 *μ*m thick paraffin sections. After rehydrating, stain the sections with H&E and Sirius red. Observe the samples with the assistance of an inverted phase contrast microscope (Olympus Corporation, Japan).

### 2.8. Rhodamine-Phalloidin Staining

Cardiomyocytes were fixed with 4% paraformaldehyde and then permeabilized with 0.5% Triton X-100. After being washed three times, cells were incubated with TRITC-phalloidin (Solarbio, CA1610) in the dark for 30 min. Also after being washed three times, cells were then counterstained with DAPI solution (Solarbio, C0060, 100 nM) for 30 s. Finally, the sections were sealed and observed under a microscope.

### 2.9. Immunohistochemistry Staining

Paraffin-sections were prepared according to the method described above. After being dewaxed, antigen retrieval was performed. The membranes were then permeabilized, and endogenous peroxides were eliminated. Sections being blocked with 10% BSA, the antibody against GSDMD, were used to incubate tissue sections overnight at 4°C. Wash tissue sections with PBS thrice, and then incubate them with anti-mouse IG-HRP at room temperature for 1 h. After being washed thrice, the sections were incubated with HRP substrate diaminobenzidine, followed by hematoxylin counterstaining. Finally, sections were dehydrated and sealed for a permanent vision under a microscope.

### 2.10. ELISA

We chose ELISA kits (Bioswamp, RA20020 and MU30369) to detect the contents of IL-1*β* in supernatant or serum. Assays were carried out under the direction of attached protocols strictly. Absorbance at 450 nm of each well was gauged by visualization of color intensity development.

### 2.11. Lactate Dehydrogenase (LDH) Assay

Plasma membrane damage was evaluated by the quantification of released LDH. The level of LDH in the supernatant was detected using an LDH cytotoxicity assay kit (Beyotime, C0016) according to the manufacturer's instructions.

### 2.12. Statistical Analysis

The data are all presented as the mean ± SEM. GraphPad Prism 8.0 (GraphPad, San Diego, CA) was used to perform statistical analysis. After the test of normality checked by the Shapiro–Wilks test and homogeneity test of variance by Levene's test, Student's *t*-test or one-way ANOVA followed by multiple comparisons test with the Bonferroni correction was carried out. *P* < 0.05 was regarded as statistical significance. All experiments were independently duplicated at least three times.

## 3. Results

### 3.1. Oridonin Can Alleviate Ang II-Induced Myocardial Hypertrophy

**Figure 1:**
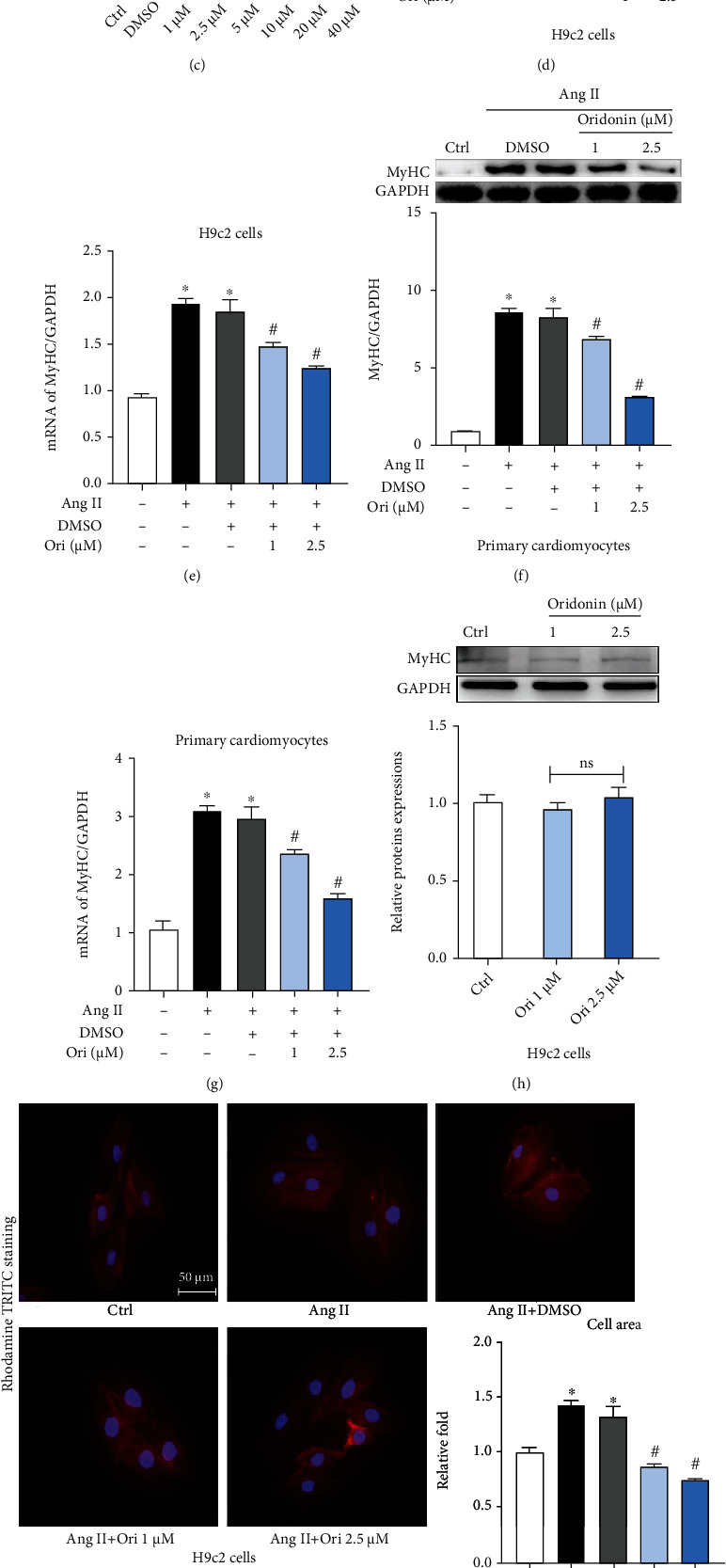
Oridonin can alleviate Ang II-induced myocardial hypertrophy. (a) Structural formula of oridonin. (b, c) The effect on cell viability after incubation of different concentrations of oridonin in H9c2 cells and neonatal rat cardiomyocytes. (d, f) Pretreated H9c2 cells and neonatal rat cardiomyocytes with Ori (1 and 2.5 *μ*M) for 1 h before daylong incubation with 1 *μ*M Ang II. Lysates were analyzed by western blotting for the expression of proteins associated with hypertrophy, and the corresponding semiquantitative statistical graph is shown. (e) The mRNA level of MyHC standardized by *β*-actin in H9c2 cells. (g) The mRNA level of MyHC in neonatal rat cardiomyocytes. (h) Analysis of the levels of MyHC in H9c2 cells when treated with Ori only. (i, j) Rhodamine-phalloidin staining to determine changes in cell size. 200x magnification; scale bar = 50 *μ*m. ^∗^*p* < 0.05 compared with control; ^#^*p* < 0.05 compared with Ang II.

The structural formula of oridonin is shown in Figure 1(a). First, we investigated the impact on the viability of cardiomyocytes when the cells were incubated with concentration gradient of oridonin for 24 h. A CCK-8 test showed an almost negligible change on cell viability both in H9c2 cells and neonatal rat cardiomyocytes when oridonin was used at low concentrations (Figures 1(b) and 1(c)). After referring to the relevant reported literature [27] and the above experimental results, two suitable concentrations were selected. We found that H9c2 cells stimulated with Ang II could exacerbate cell hypertrophy, while oridonin inhibited this effect in a concentration-dependent manner (Figure 1(d)). Similarly, the effect of oridonin to relieve myocardial hypertrophy has been confirmed at the mRNA level (Figure 1(e)). However, there was no significant effect on H9c2 cells when only oridonin was used (Figure 1(h)).

The fluorescence staining of the ghost ratio cyclopeptide also showed the same result (Figure 1(i)). We then repeated the above experiments in neonatal rat cardiomyocytes, and we found that oridonin fought myocardial hypertrophy not only in H9C2 cells but had similar effects in neonatal rat cardiomyocytes. Oridonin relieved Ang II-induced cardiomyocytes hypertrophy and reduced MyHC expression both in protein and mRNA levels (Figures 1(f) and 1(g)). The fluorescence staining of the ghost ratio cyclopeptide in neonatal rat cardiomyocytes also showed that oridonin could effectively alleviate the occurrence of cardiomyocyte hypertrophy (Figure 1(j)).

### 3.2. Ang II-Induced Inflammation Was Inhibited by Oridonin

**Figure 2:**
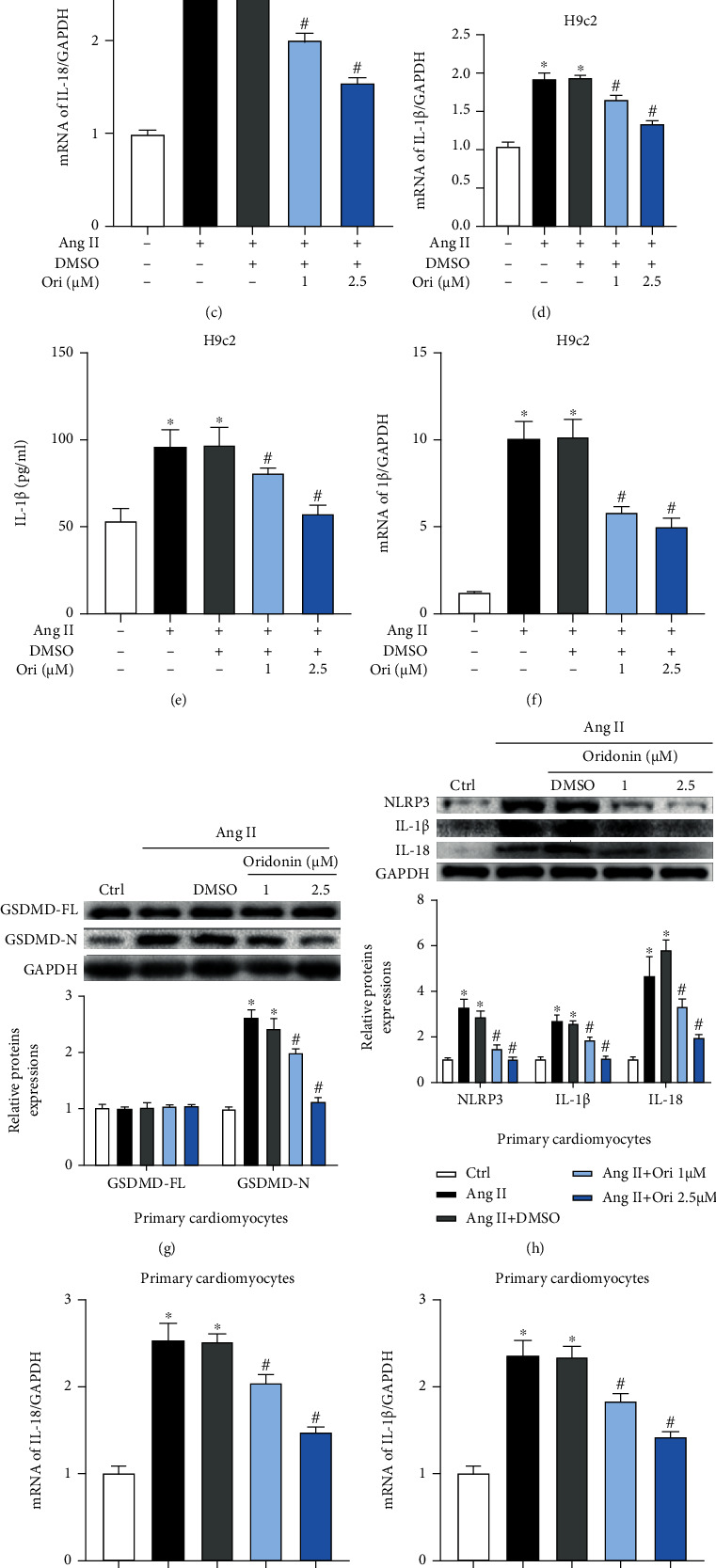
Ang II-induced inflammation was inhibited by oridonin. Detection of GSDMD-mediated inflammation levels after being incubated with Ang II daylong and pretreated with different concentrations of Ori for 1 h. (a) Gel images of GSDMD and its relevant statistical graph in H9c2 cells. (b) Gel images of NLRP3, IL-1*β*, and I-18 and its relevant statistical graph in H9c2 cells. (c, d) The mRNA level of IL-18 in H9c2 cells. (e, f) Contents of IL-1*β* and LDH in the supernatant in H9c2 cells. (g) Gel images of GSDMD and its relevant statistical graph in neonatal rat cardiomyocytes. (h) Gel images of GSDMD-related inflammation factors and its relevant statistical graph in neonatal rat cardiomyocytes. (i, j) The mRNA level of IL-18 and IL-1*β* in neonatal rat cardiomyocytes. (k, l) Contents of IL-1*β* and LDH in the supernatant in neonatal rat cardiomyocytes. ^∗^*p* < 0.05 compared with control; ^#^*p* < 0.05 compared with Ang II.

In this section, we were surprised to find that Ang II can induce GSDMD-related inflammatory response in cardiomyocytes through 24 hours of stimulation. We then analyzed the correlation between oridonin and GSDMD-mediated inflammation. H9c2 cells incubated with Ang II; GSDMD-N levels at both concentrations of oridonin decreased compared with those of the Ang II group (Figure 2(a)). We then detected the expression of related inflammatory factors and found that the upstream inflammatory factor NLRP3 of GSDMD increased under the stimulation of Ang II, while its expression decreased significantly after the treatment of oridonin (Figure 2(b)). Similarly, the levels of IL-1*β* and IL-18 (Figures 2(b)–2(d)) after treatment with oridonin were cut down compared with the Ang II group in both protein and mRNA levels. The determination of IL-1*β* and LDH in the supernatant also showed the same results (Figures 2(e) and 2(f)).

We also further demonstrated that Ang II can induce GSDMD-related inflammatory responses in neonatal rat cardiomyocytes. The application of oridonin reduced GSDMD activation (Figure 2(g)) and cut down the level of NLRP3, IL-1*β*, and IL-18 in neonatal rat cardiomyocytes (Figures 2(h)–2(k)), as well as the release of LDH (Figure 2(l)).

Therefore, we conclude that Ang II can stimulate cardiomyocytes to activate GSDMD related inflammatory response leading to the increase of related inflammatory factors and extracellular IL-1*β* and LDH release. The application of oridonin can alleviate this inflammatory reaction.

### 3.3. Inhibition of GSDMD Alleviated Hypertrophy Caused by Ang II

**Figure 3:**
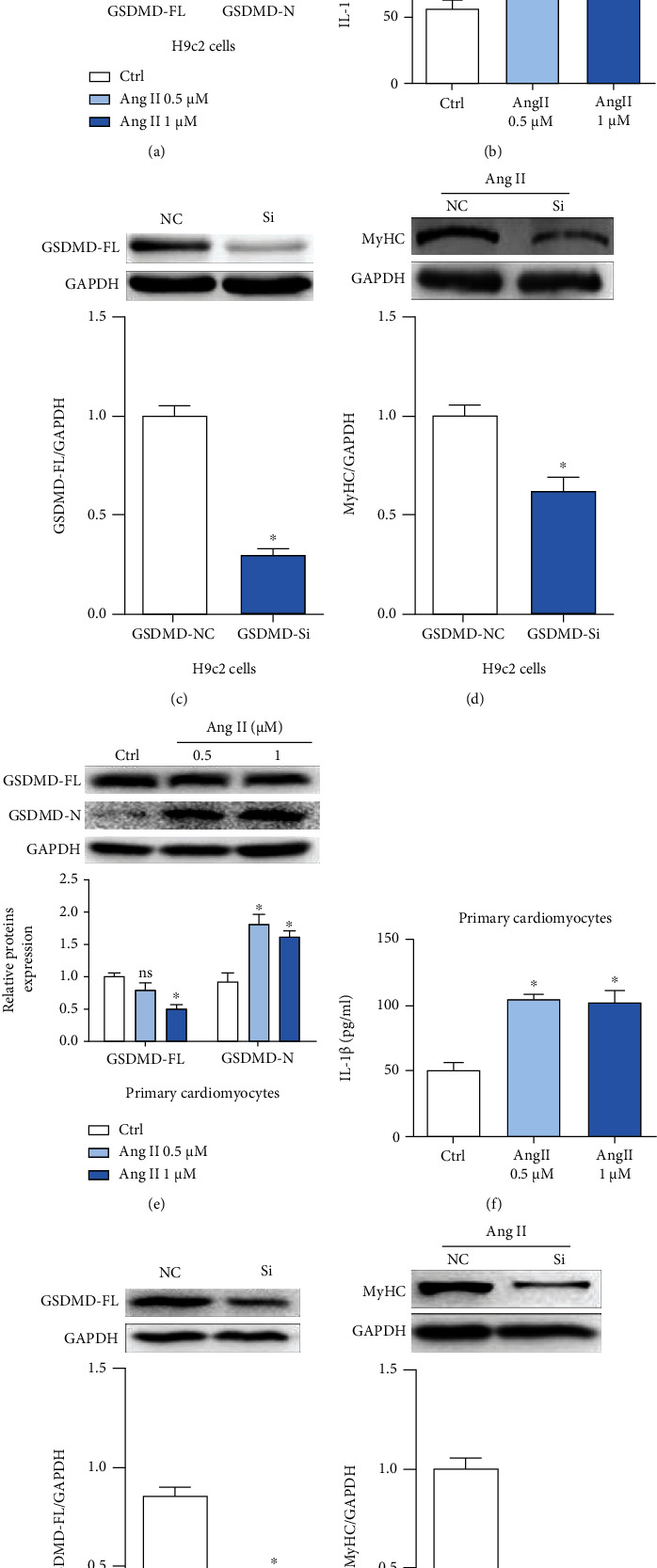
Inhibition of GSDMD alleviated hypertrophy caused by Ang II. (a, e) Gel images of GSDMD-FL and GSDMD-N in H9c2 and neonatal rat cardiomyocytes incubated with Ang II (0.5, 1 *μ*M); the semiquantitative statistical graph is shown. (b, f) The supernatant in IL-1*β* levels gauged by ELISA kits. (c, g) Gel images of GSDMD after knockdown by siRNA; the relevant statistical graph is shown. (d, h) Levels of hypertrophy indicator MyHC were detected in cell lysates after knockdown by siRNA, and the relevant statistical graph is shown. ^∗^*p* < 0.05 compared with control; ^#^*p* < 0.05 compared with Ang II.

Previous studies have shown that cardiac remodeling resulting from the induction of Ang II can be alleviated in NLRP3^−/−^mice [28]. Moreover, the inflammasome mainly formed by NLRP3 is the key priming factor in the classical pathway of pyroptosis. Thus, we explored the relationship between Ang II and GSDMD, key protein in pyroptosis [29, 30]. Two concentrations were selected (0.5 and 1 *μ*M) according to the preliminary results. After being stimulated with Ang II, we can see that the expression of GSDMD-N was upregulated in H9c2 cells (Figure 3(a)). Furthermore, the expression of IL-1*β* in the culture supernatant was also upregulated (Figure 3(b)). These results suggested some kind of relevancy between Ang II and GSDMD-mediated inflammation.

To confirm this conjecture, we constructed small interfering RNAs to silence the expression of GSDMD (Figure 3(c)). After incubation, the level of hypertrophy associated protein MyHC was obviously lower compared with that in the NC group (Figure 3(b)), consistent with our previous conjecture.

To further verify the conjecture, we repeated the above experiments on neonatal rat cardiomyocytes and obtained highly consistent experimental results (Figures 3(e)–3(h)). It was observed that Ang II could induce GSDMD-related inflammatory responses in both H9c2 cells and neonatal rat cardiomyocytes. And after inhibiting GSDMD, Ang II-induced cardiac hypertrophy was significantly alleviated.

### 3.4. Oridonin Attenuated Cardiac Remodeling by Inhibiting GSDMD-Mediated Inflammation In Vivo

**Figure 4:**
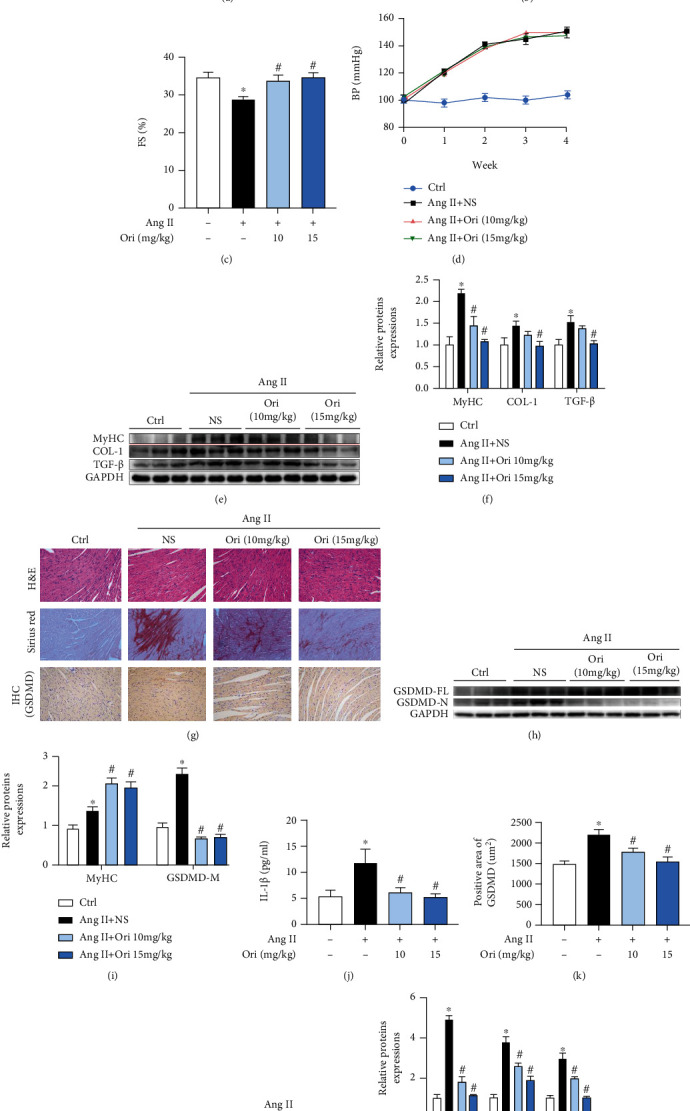
Oridonin attenuated myocardial cardiac remodeling by inhibiting GSDMD-mediated inflammation in vivo. The mice were examined with an echocardiogram at the end of the animal experiment. (a) Representative cardiac echocardiographic images of the four groups of mice. (b, c) EF and FS values for the four groups. (d) Changes in blood pressure in mice of each group during modeling. (e, f) Hypertrophy and fibrosis levels in cardiac tissue were detected by western blotting. (g) Representative H&E and Sirius red staining of each group also showed disordered myocardial cell structure and fibrosis in cardiac tissue. (g–i) The results of IHC showed the different expression levels of GSDMD in cardiac tissue corresponding to the results of western blot. (j) The content of IL-1*β* in serum. (k) The relevant semiquantitative statistical graph of GSDMD-IHC. (l, m) Gel images of NLRP3, IL-1*β*, and IL-18 in myocardial tissue and its semiquantitative statistical graph. ^∗^*p* < 0.05 compared with control; ^#^*p* < 0.05 compared with Ang II. EF: ejection fraction; FS: fractional shortening.

The above results were further confirmed in vivo. The results of cardiac Doppler ultrasound of the mice showed that the cardiac function of mice infused with Ang II was significantly reduced, while the EF and FS values were alleviated after oridonin treatment (Figures 4(a)–4(c)). The blood pressure of the mice increased significantly after infusion of Ang II, but treatment with oridonin had no effect on it (Figure 4(d)). Meanwhile, the incremental levels of myocardial hypertrophy and fibrosis were neutralized by oridonin (Figures 4(e) and 4(f)), and the high concentration showed a more significant effect. Morphological disorder and collagen fiber deposition of myocardial cells in the model group were seen by HE staining of paraffin tissue sections of the heart, and these effects were improved after the application of oridonin (Figure 4(g)). Sirius red staining also showed similar results (Figure 4(g)). The red area of fiber tissue increased significantly after Ang II infusion, while the degree of fibrosis was effectively alleviated after the application of oridonin. Consistent with the cell results, Ang II activated cell inflammation and caused myocardial remolding in mice, showing increasing contents of GSDMD-N and IL-1*β* in serum (Figures 4(g)–4(k)). The same results were obtained from the protein semiquantitative analysis of GSDMD-related inflammatory factors in myocardial tissue (Figures 4(l) and 4(m)). The use of oridonin effectively alleviated Ang II-induced fibrosis and inflammation.

## 4. Discussion

Ang II signaling is the most extensively studied mechanism of cardiac hypertrophy and fibrosis. Also, Ang II can directly promote cardiomyocytes to grow in vitro [31]. In this study, we validated that Ang II could promote hypertrophy in cardiomyocytes, consistent with the results from previous investigations. Also, the anti-inflammation drug oridonin alleviated cardiac remodeling caused by Ang II as expected.

In the process of myocardial remodeling, inflammation promotes the development of myocardial remodeling, and the correlation between the two has been confirmed by a host of studies [32, 33]. Many proinflammatory factors take part in this process, including IL-1*β* [34], IL-18 [5], NF-*κ*B [6, 35], and TLR4 inhibitor signal pathway [36] as well as increased ROS formation due to the engagement of Nox2 [37] and Nox4 [38]. Notably, an anti-inflammatory drug called pentoxifylline was found to have an influence on attenuating cardiac fibrosis and hypertrophy in rats treated with Ang II [39]. This report suggests that it is feasible to alleviate the occurrence of myocardial remodeling by inhibiting inflammatory response. Oridonin, which is also an anti-inflammatory drug, may have similar effects.

Nowadays, with inflammation-related protein-GSDMD getting into act, various effects of GSDMD have been emerging. At the same time, because GSDMD-mediated inflammation is strongly coupled with NLRP3 inflammasome and Ang II has shown a certain correlation with NLRP3 in many studies, we tried to explore the potential relationship between Ang II-induced cardiac remodeling and GSDMD-mediated inflammation. We found that under the treatment of Ang II, GSDMD was activated and cut into the active structure GSDMD-N, along with an increase of NLRP3 and GSDMD-related inflammation factors, IL-1*β* and IL-18, in both protein and mRNA levels. The release of extracellular LDH also increased. The results showed that with the stimulation of Ang II, the process of cardiomyocyte hypertrophy was accompanied by the increase of GSDMD-mediated inflammatory response. The same results have been found in animal experiments. Therefore, we speculated that Ang II might aggravate hypertrophy in cardiomyocytes by GSDMD-mediated inflammation. With the utilization of siRNA, we found that hypertrophy induced by Ang II decreased in H9c2 cells with specific inhibition of GSDMD. These results suggested that GSDMD might play a vital part in cardiac remodeling induced by Ang II. In subsequent experiments, we are supposed to use GSDMD knockout mice to authenticate it further.

As a widely used anti-inflammatory drug, our results showed that oridonin could also effectively alleviate GSDMD-related inflammatory response. Our data showed that oridonin presented a good inhibitory effect on GSDMD-mediated inflammation both in vitro and in vivo. Meanwhile, oridonin also alleviated the occurrence of Ang II-induced myocardial remodeling. Since we have confirmed that inhibition of GSDMD can significantly alleviate the process of myocardial remodeling, we finally reach the conclusion that oridonin improved cardiac remodeling through inhibiting GSDMD-mediated inflammation. However, the specific mechanism has not been further discussed. Further experiments will be supposed to explore the essential causes of this phenomenon and its possible mechanism. Also, it is worth noting that previous studies have shown that Ang II can act independently of, or in synergy with, increased blood pressure to induce cardiac remodeling [40, 41]. Our study showed that the application of oridonin did not change the blood pressure of mice, suggesting that its mechanism of alleviating myocardial remodeling may not be related to blood pressure, but whether it is related to AT1R-associated protein [42] needs further experimental verification. Besides, studies have shown that oridonin can also exert anti-inflammatory effects by inhibiting NF-*κ*B signaling pathway [43, 44]. At the same time, oridonin can inhibit the production of ROS and oxidative stress by inhibiting mitochondrial dysfunction. Whether oridonin can alleviate myocardial remodeling through other ways other than GSDMD remains to be further verified.

**Figure 5:**
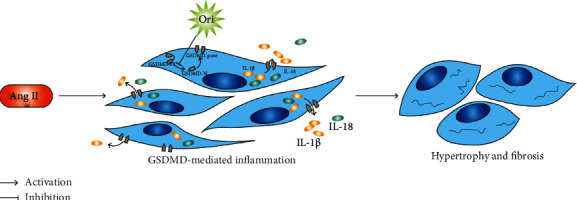
The mechanism schematic of oridonin attenuating cardiac remodeling by inhibiting GSDMD-mediated inflammation.

Altogether, we confirmed that oridonin could alleviate Ang II-induced cardiac remodeling by inhibiting GSDMD-mediated inflammation (the mechanism is shown in Figure 5). As a widely used anti-inflammatory drug, oridonin is expected to have great prominence in new fields.

## Figures and Tables

**Table 1:** Relative primer sequence used in real-time PCR.

Target	Primer	Sequence
Myh7 (rat)	Forward	5′-CCAGAACACCAGCCTCATCAACC-3′
	Reverse	5′-CACCGCCTCCTCCACCTCTG-3′
IL-1*β* (rat)	Forward	5′-CTCACAGCAGCATCTCGACAAGAG-3′
	Reverse	5′-TCCACGGGCAAGACATAGGTAGC-3′
IL-18 (rat)	Forward	5′-CGACCGAACAGCCAACGAATCC-3′
	Reverse	5′-TCACAGATAGGGTCACAGCCAGTC-3′
GAPDH (rat)	Forward	5′-CCGCATCTTCTTGTGCAGTG-3′
	Reverse	5′-GAGAAGGCAGCCCTGGTAAC-3′

## Data Availability

The authors confirm that the data supporting the results of this study can be found in the article.
